# Normative Observational Nerve Ultrasound Values in School-Age Children and Adolescents and Their Application to Hereditary Neuropathies

**DOI:** 10.3389/fneur.2020.00303

**Published:** 2020-04-28

**Authors:** Anna-Sophie Grimm, Charlotte Schubert, Alexander Grimm, Jan-Hendrik Stahl, Hanna Küpper, Veronka Horber, Josua Kegele, Sophia Willikens, Julia Wittlinger, Lina Serna-Higuita, Natalie Winter, Samuel Groeschel

**Affiliations:** ^1^Department of Pediatric Neurology, University Children's Hospital Tübingen, Tübingen, Germany; ^2^Department of Neurology and Hertie Institute for Clinical Brain Research (HIH), University of Tübingen, Tübingen, Germany; ^3^Center of Neurology and Hertie Institute for Clinical Brain Research (HIH), University of Tübingen, Tübingen, Germany; ^4^University Hospital Tübingen, Neurology, Tübingen, Germany; ^5^Department of Clinical Epidemiology and Applied Biostatistics, Tübingen University, Tübingen, Germany

**Keywords:** nerve ultrasound, children neuropathy, reference values, lysosomal storage diseases, nerve imaging

## Abstract

**Backgrounds:** We have aimed to establish nerve ultrasound reference data in 8 to 17-year-old children and adolescents and to compare those data to younger children, adults, and age-matched children with polyneuropathies.

**Methods:** High-resolution ultrasounds of the nerves were performed in 117 healthy children and adolescents at 20 predefined landmarks in the neck and the extremities of both sides. Mean values, side-to-side differences and intraneural ratios, as well as upper limits have been calculated. In a second step, a comparison between 25 children and adolescents of the same age range with proven hereditary and acquired neuropathies and lysosomal storage diseases has been carried out.

**Results:** Nerve growth correlates significantly with age and reaches adult values at the age of around 15 years. The influence of body mass index and gender is negligible at most segments. By the use of age-specific upper limits, nerve enlargement could be seen in distinct types of neuropathies, particularly in demyelinating hereditary and inflammatory types, which is comparable to findings in adults, but also in rare lysosomal storage diseases.

**Conclusion:** Nerve size correlates with age during childhood and reaches a climax in younger adults. Age-matched reference data are inevitable to differ between hypertrophic and non-hypertrophic nerve damage, e.g., in neuropathies.

## Introduction

Nerve imaging in nerve pathologies has recently become a main focal point. By the use of magnetic resonance neurography and high-resolution ultrasonography, more knowledge about nerve morphology has been established. In HR-US, nerve enlargement due to tumors, neuromas, or inflammation has been the most profound finding (1–4). Thus, normal values for distinct nerves and locations are essential (5–10). As we hypothesized that distinct nerve values in school age children and adolescents exist compared to adults, we thus analyzed healthy individuals aged between 8 and 17 years and compared their values to epidemiological data. Finally we compared the collected normal data to children and adolescents with a proven diagnosis of assumed hypertrophic and non-hypertrophic neuropathies.

## Methods

Healthy children/adolescents of 8 to 17 years of age were recruited from schools in Tuebingen County between May 2018 and 2019. The study was approved by the local ethics committee (Tuebingen 765/2017BO1). The informed consent was explained to the probands and signed by a parental authority. A medical questionnaire containing medical history, developing mile stones, family medical history, height, weight, gender, ethnicity, and handedness was performed next, followed by a short neurological examination. Exclusion criteria were confounding diseases, such as known untreated diabetes mellitus.

In a second step, ultrasound results of 25 children and adolescents of the same age range with a definite diagnosis of acquired or inherited polyneuropathy were analyzed. Statistical comparisons to the reference group were not carried out due to the small sample size of the groups. Informed consent was retrospectively obtained.

### Ultrasound Examination

Nerve ultrasounds were carried out by CS and ASG blinded to each other by the use of a high resolution linear transducer (14 MHz, Mindray TE7, Ultrasound systems, Darmstadt, Germany). The authors were orientated on protocols, and they had previously performed other studies involving adults (9, 11–14). In all children, arm and leg nerves of the right side were examined at several locations: the median and ulnar nerve (MN, UN) at the mid-upper arm, the elbow, and the mid-forearm as well as the MN at the wrist. The radial nerve was measured at the Frohse arcade, and its superficial branch and the posterior interosseous nerve (PIN) were also analyzed. The fibular and tibial nerve (FN, TN) were screened at the popliteal fossa and the ankle (i.e., the fibular superficial nerve, FSN). Furthemore, the sural nerve was measured at the distal calf next to the vein. The roots C5 and C6 were measured laterally to the transverse process, and the vagus nerve was measured at the level of the carotid triangle. On the left side, the MN upper arm segment, the TN at the ankle, and root C5 were measured. Here, we also calculated the percentage of the side-to-side difference.

In all nerves, the cross-sectional area was measured (CSA), except for the roots, which were measured in a long-axis view as diameter. The measurement was carried out just inside the hyperechoic rim of the nerve (13).

Furthermore, we calculated the intranerve-CSA-variability (7) in those nerves, with more than one landmark measured, i.e., the MN, UN, and TN.

### Statistical Analyses

Descriptive statistics were used to describe the sample, and for some statistical analyses two groups were built (Group A children were aged between 8 and 12 years and group B adolescents between 13 and 17 years). Continuous variables were analyzed either as mean and standard deviation (±SD) or as median and interquartile range (IQR) depending on the distribution of the data. Normal distribution was assessed by investigating kurtosis, skewness, histograms, and Q-Q plots. Categorical variables were described as percentages and absolute frequencies. In addition, to determine the reference ranges of the assessed nerves, we calculated the 95% confidence intervals, defined as the interval between which 95% of the values of a reference population fall into.

A bivariate analysis was performed to compare baseline characteristics. A Chi square test for categorical variables was used. Independent-sample *T*-tests were used to compare numerical variables that were approximately normally distributed, whereas Mann-Whitney tests were used to evaluate non-normally distributed variables.

The intranerve variability was calculated as the ratio between maximum CSA and minimum CSA. The side-to-side difference was calculated as the difference between the right and left CSA. A dependent-sample t test was used to find out whether these variables were normally distributed, while a Wilcoxon Test was used to evaluate non-normally distributed variables. The percentage of the side-to-side difference was calculated as the ratio of this difference vs. the right CSA. The relationship between CSA with demographic parameters was evaluated using an analysis of covariance (ANCOVA), with age, BMI, and gender being the independent variables. Several assumptions were tested before the model was carried out: normal distribution of residual, homocedasticity, and homogeneity of variance. The intrarater and interrater intraclass correlation coefficient (ICC) was calculated through the use of post-processing.

The percentile curves of the nerves were calculated by the LMS method using R's VGAM package version. The LMS method plots the shapes of the percentiles by three uncorrelated curves: the L, M, and S curves. The first defines the skewness (L) of the distribution at each age, the second the median (M), and the third the coefficient of variation (S). The three parameters are constrained to change smoothly as the covariate changes, and they can be plotted against the covariate (15, 16). The percentile curves were plotted with qtplot, R Studio version 3.6.

All statistical tests were two-tailed, the significance level was set at *p* ≤ 0.05, and results are reported with 95% confidence intervals (95% CI). The analyses were carried out using SPSS (IBM 25 Corp, Armonk, NY) and R Software version 3.6.

## Results

Overall, 117 children/adolescents (59 8–12-year-olds and 58 13–17-year-olds) have been included. One girl had well-treated diabetes mellitus type 1 without secondary organ affection. In Group A, a non-significant slight female dominance occurred compared to Group B (*p* = 0.519); furthermore, more children were left-handed in this group (9:4, Fishers exact *p* < 0.05). As expected, the BMI in group B was higher than in group A (*p* < 0.001) (see Table 1). All measured segments were normally distributed except for the root C5 and C6 values. Most measurement points have been analyzed in all subjects; however, C5 and C6 values had to be excluded by post-processing imaging in some children/adolescents, particularly in the younger group, due to restricted cooperation and scanning angle. The grouped mean overall CSA values and standard deviations (as well asmedian and IQR, particularly for C5 and C6) are shown in Table 1. The inter- and intrarater ICC was >0.9 for both examiners.

**Table 1:** Baseline characteristics of the study population by age-groups (group A: children 8–12 years old and group B: adolescents 13–17 years) (*n* = 117).

	***n***		**Group A (*n* = 59)**		**Group B (*n* = 58)**		***P*-value**
Gender Female n (%) Male n (%)	117		32 (54.2%) 27 (45.8%)		28 (48.3%) 30 (51.7%)		0.519^*^
BMI mean (±SD)	115		16.21 (±1.99)		20.90 (±3.19)		<0.001^**^
Weight in Kg mean (±SD)	115		33.15 (±6.39)		62.82 (±13.21)		<0.001^**^
Height in cms mean (±SD)	117		1.42 (±0.09)		1.73 (±0.09)		<0.001^**^
**Nerve CSA values**		**Group A (*****n*** **=** **59)**			**Group B (*****n*** **=** **58)**		
		**mean (**±**SD)**	**95% CI**	**median (p25–75)**	**mean (**±**SD)**	**95% CI**	**median**
MN-UA	117	6.78 (±1.63)	6.36–7.20	7.0 (6.0–8.0)	9.88 (±2.58)	9.20–10.56	10.0 (8.0–12.0)
MN-elbow	117	5.85 (±1.55)	5.44–6.25	6.0 (5.0–7.0)	8.81 (±2.87)	8.06–9.56	8.0 (7.0–10.0)
MN-FA	117	5.44 (±1.32)	5.10–5.78	5.0 (5.0–6.0)	7.17 (±2.09)	6.62–7.72	7.0 (6.0–9.0)
MN-wrist	116	7.12 (±1.54)	6.74–7.49	7,0 (6.0–8.0)	9.52 (±2.30)	8.91–10.12	9.5 (8.0–11.0)
UN-UA	117	4.75 (±1.37)	4.39–5.10	4.0 (4.0–6.0)	6.66 (±2.07)	6.11–7.20	6.0 (5.0–8.0)
UN-elbow	116	2.93 (±1.56)	2.53–3.34	2.0 (2.0–4.0)	5.26 (±2.35)	4.64–5.89	5.0 (3.0–7.0)
UN-FA	117	3.95 (±1.24)	3.63–4.27	4.0 (3.0–5.0)	5.50 (±1.68)	5.06–5.94	5.0 (4.0–6.3)
RN-S	117	1.07 (±0.25)	0.87–1.47	1.0 (1.0–1.0)	1.59 (±0.56)	1.25–1.93	1.5 (1.0–2.0)
RN-PIN	117	1.39 (±0.53)	1.25–1.53	1.0 (1.0–2.0)	1.97 (±0.70)	1.78–2.15	2.0 (1.8–2.0)
TN-PF	103	19.6 (±4.74)	18.3–20.8	20 (16–23)	25.7 (±7.36)	23.51– 27.8	25 (20–33)
TN-ankle	116	6.24 (±1.92)	5.74–6.75	6.0 (5.0–7.0)	8.57 (±2.02)	8.04–9.10	8.0 (7.0–9.3)
PN-PF	106	3.61 (±1.88)	3.12–4.11	3.0 (2.0–4.0)	6.06 (±2.59)	5.32–6.81	6.0 (4.0–8.0)
PN sup	116	2.46 (±1.13)	2.16–2.75	2.0 (2.0–3.0)	3.12 (±1.35)	2.76–3.48	3.0 (2.0–4.0)
SN	117	1.24 (±0.47)	1.14–1.95	1.0 (1.0–1.0)	1.84 (±0.65)	1.45–2.21	2.0 (1.0–2.0)
VN	117	1.42 (±0.50)	1.29–1.55	1.0 (1.0–2.0)	2.05 (±0.61)	1.89–2.21	2.0 (2.0–2.0)
C5^***^	67	2.00 (±0.32)	1.86–2.15	2.0 (1.8–2.3)	2.02 (±0.38)	1.90–2.13	2.0 (1.8–2.3)
C6^***^	54	2.80 (±0.49)	2.50–3.10	2.7 (2.4–3.3)	2.80 (±0.53)	2.63–2.96	2.8 (2.3–3.2)

* Chi quadrat Test,

** Independent samples T-Test, SD: standard deviation, CI: confidence interval.

*** All values (except for C5 and C6) are in mm^2^. Most nerve segments (except for C5 and C6) are significantly smaller in the younger children group than in adolescents (p < 0.01). The left-side values are: MN-UA (6.33 ± 1.66 and 9.56 ± 2.58, TN-ankle* 6.32 ± 1.91 and 8.51 ± 2.01, C5* 2.01 ± 0.31 and 2.09 ± 0.34). Ninety-fifth percentage, Due to practicability the grouped mean values are shown here.

### CSA Values in School Children and Adolescents

**Figure 1:**
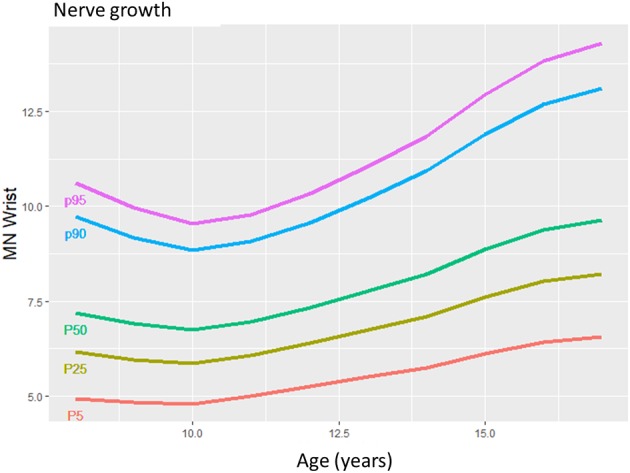
The mean nerve growing in children from 8 to 17 years in a selected median nerve segment. The more prominent growth during puberty compared to younger children is seen by the slope of the lines.

The nerve CSA correlated significantly with age in all measured segments (see Table 2 and Figure 1). Concerning BMI and CSA, significant correlations were only seen in the distal median nerve segments (MN-elbow and MN-FA *p* < 0.05). Boys showed higher CSA values in the median nerve and the proximal tibial nerve but not elsewhere (Table 2). The right median nerve was significantly larger than the left at the upper arm (*p* < 0.05). This side preference, however, was not found in the case of the tibial nerve and root C5 (*p* > 0.05). The percentage of the side-to-side difference of the MN, the TN, and root C5 did not show significant differences between the age groups (*p* > 0.05), ranging between 1 and 5% each nerve in the mean (SD up to 30%). The intranerve–CSA variability was 1.36 (SD 0.38) in the MN, 1.25 in the UN (SD 0.34), and 3.23 (SD 1.11) in the TN. Between group A and B, no significant differences were found (*p* > 0.05).

**Table 2:** The relationship between BMI, age, gender and the CSA of nerves (ANCOVA).

	**B**	**95% CI**	***P*-value**
**MN-UA**			
Gender male	1,086	0,328–1,844	**0,005**
Alter	0,396	0,230–0,562	**<0,001**
BMI	0,098	−0,057–0,253	0,212
**MN-elbow**			
Gender male	0,811	0,030–1,592	**0,042**
Alter	0,372	0,200–0,543	**<0,001**
BMI	0,176	0,016–0,335	**0,032**
**MN-FA**			
Gender male	0,720	0,133–1,307	**0,017**
Alter	0,149	0,021–0,278	**0,023**
BMI	0,177	0,057–0,297	**0,004**
**MN-wrist**			
Gender male	0,477	−0,253–1,206	0,198
Alter	0,323	0,162–0,483	**<0,001**
BMI	0,067	−0,082–0,216	0,375
**UN-UA**			
Gender male	0,516	−0,120–1,151	0,111
Alter	0,251	0,112–0,390	**0,001**
BMI	0,067	−0,063–0,197	0,312
**UN-elbow**			
Gender male	−0,065	−0,804–0,673	0,861
Alter	0,305	0,142–0,468	**<0,001**
BMI	0,088	−0,063–0,239	0,249
**UN-FA**			
Gender male	0,433	−0,253–1,206	0,198
Alter	0,171	0,162–0,483	**<0,001**
BMI	0,070	−0,082–0,216	0,375
**RN-PIN**			
Gender male	0,309	0,085–0,534	**0,007**
Alter	0,083	0,034–0,132	**0,001**
BMI	0,005	−0,041–0,051	0,838
**TN-PF**			
Gender male	2,878	0,582–5,173	**0,015**
Alter	0,661	0,151–1,170	**0,012**
BMI	0,414	−0,067–0,895	0,090
**TN-ankle**			
Gender male	−0,143	−0,844–0,557	0,685
Alter	0,303	0,151–0,456	**<0,001**
BMI	0,106	−0,038–0,249	0,147
**FN-PF**			
Gender male	−0,221	−1,115–0,672	0,624
Alter	0,338	0,143–0,532	**0,001**
BMI	0,060	−0,118–0,239	0,505
**FN sup**			
Gender male	0,417	−0,033–0,867	0,069
Alter	0,125	0,026–0,223	**0,014**
BMI	−0,002	−0,094–0,090	0,969
**VN**			
Gender male	0,151	−0,052–0,354	0,143
Alter	0,088	0,043–0,132	**<0,001**
BMI	0,013	−0,028–0,055	0,531

*Analysis of covariance, adjusted by gender, age and body index mass. Significant values p < 0.05 are shown in bolt print. The nerves SN, C5 and C6 cannot perform ANCOVA because they do not meet all the required assumptions*.

**Figure 2:**
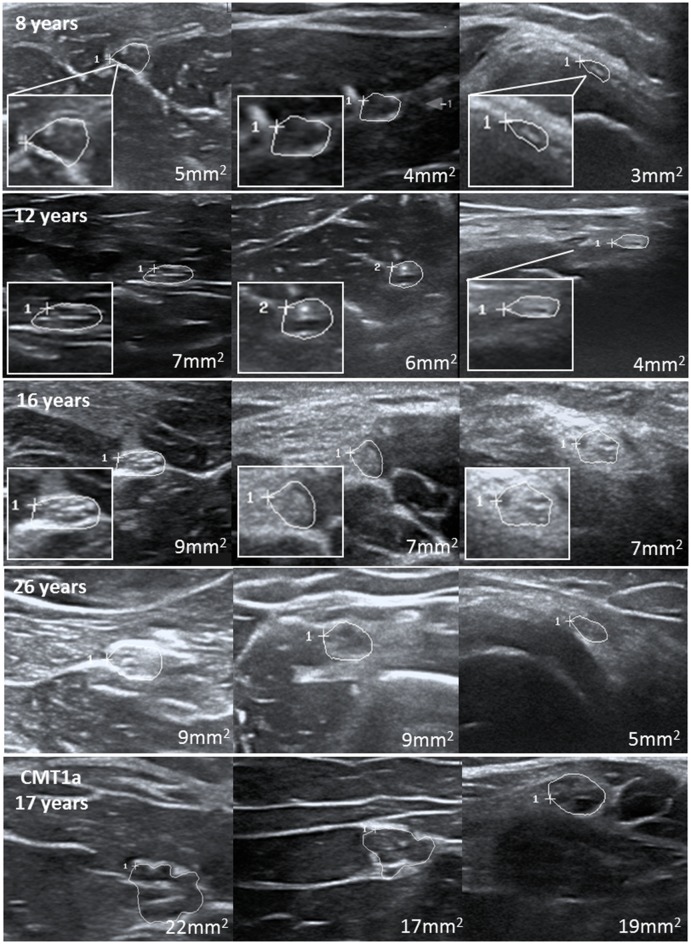
Examples of several nerves at different ages with consistent increase of nerve cross-sectional area. The last images show an example of these nerves in a teenager with CMT1b at the age of 17.

Using percentile calculation by the LMS method, we could define the percentile values for each age and each nerve segment (Table 3). Figure 2 shows several anatomical landmarks at different ages compared to one adolescent with hereditary polyneuropathy.

**Table 3:** Presumed reference values per age compared to adults from literature (upper limits = 90. Percentile, 15).

**Age in years**	**8**	**9**	**10**	**11**	**12**	**13**	**14**	**15**	**16**	**17**	**Adults**
**Nerve in mm**^2^											**(****9****)**
MN-UA	8.5	9	9	9.5	10	10.5	11.5	12	13	14	12
MN-elbow	8	8	8	8.5	8.5	9	10	11	12	13	12.5
MN-FA	7	7	7	7.5	7.5	8	8.5	9	9.5	10	10
MN-wrist	9	9	9.5	9.5	10	10.5	11	11.5	12	13	16.5
UN-UA	6.5	6.5	6.5	6.5	7	7.5	8	8.5	9	9.5	9.5
UN-elbow	5	5	5	5	5.5	6	6.5	7.5	8.5	9.5	13
UN-FA	5.5	5.5	5.5	6	6.5	6.5	7	7	7.5	7.5	8.5
RN-S	1.5	1.5	1.5	1.5	2	2	2	2.5	2.5	2.5	3
RN-PIN	2	2	2	2	2.5	2.5	3	3	3	3	3
TN-PF	25	26	26	27	28	29	31	32	34	36	33^**^
TN-ankle	9	9	9	9	9	10	10.5	10.5	10.5	11	14
FN-PF	6	6	6	6	6.5	7	8	9	10	11	11.5
FN-S	2.5	2.5	2.5	3	3	3	3.5	3.5	3.5	4	3.5
SN	2.5	2.5	2.5	2.5	2.5	3	3	3	3.5	3.5	3.5
VN	2.5	2.5	2.5	2.5	2.5	2.5	2.5	3	3	3.5	3.5
C5^*^	2.7	2.7	2.7	2.7	2.7	2.8	2.8	2.9	2.9	2.9	2.9
C6^*^	3.9	3.9	3.9	3.9	4.0	4.0	4.1	4.1	4.2	4.2	4.2

Normal values at each segment and each age in mm^2^.

* For the spinal nerves C5 and 6 the values are in millimeter.

** *male >65 years 37 mm^2^*.

### Comparison to Age-Matched Patients With Ascertained Polyneuropathy

At last, we compared our upper limits and normal values to children with distinct neuropathies. Overall, 25 patients aged between 8 and 18 years with distinct diagnoses have been included. Ultrasound examinations were carried out using the same device and mostly the same protocol, except for some segments in some patients. All children and adolescents with genetically proven Charcot Marie Tooth neuropathy (CMT) 1a (gene: PMP 22 duplication), CMT1b (MPZ), CMT4c (SH3TC2), and CMT4d (NDRG1), which are all hereditary neuropathies with demyelination; were also revealed to have enlarged nerves, the majority of them present in most segments compared to the age-matched reference values. In contrast, axonal variants, i.e., CMT2, showed no nerve enlargement. Further demyelinating neuropathies, in detail lysosomal storage diseases [metachromatic leukodystrophy (MLD), globoid cell leukodystrophy (Morbus Krabbe), and multiple sulfatase deficiency (SUMF1 gene)] also showed multifocal nerve enlargement. A patient with very rare inherited neuropathy (giant axonopathy, gigaxonin gene) revealed also nerve enlargement, whereas those patients without neuropathy (one with diffuse pain syndrome and one with cognitive deficits) revealed normal nerve values. Inflammatory neuropathies showed multifocal proximal predominant nerve enlargement with involvement of upper arm segments in CIDP and root/vagus predominance in GBS. Two patients with neurofibromatosis type 1 have also been analyzed, of whom one showed multiple nerve enlargements due to nerve tumors, and the other showed overall normal segments due to missed tumors. Table 4 summarizes all nerve measurements of these children/adolescents. An example of significantly enlarged nerve segments in comparison to normal nerves is shown in Figure 2.

**Table 4:** Children and adolescents with polyneuropathy.

**Patient**	**Age**	**Diagnosis**	**MN** **(OA, elbow, FA wrist)**	**UN** **(OA, elbow, FA)**	**RN** **(sup, PIN)**	**TN** **(pop, distal)**	**PN** **(pop, distal)**	**SN**	**C5, C6**	**VN**	**Enlargement**
		Hereditary neuropathies									
1	14	***CMT1a***	11/9/8/5	8/**9/**7	NA/2	**32/12**	**9**/2	2	2.5/2.6	1	**4/16**
2	18^*^	**CMT1a**	**26/25/20/13**	**22/13/17**	**4/3**	**84/25**	**28/4**	**6**	**3.3/4.5**	**4**	**17/17**
3	18^*^	**CMT1a**	**32/21/15/13**	**17/11/13**	**5/4**	**61/25**	**21/4**	**5**	**3.6/5.9**	**4**	**17/17**
4	9	**CMT1a**	**9/12/NA/9**	**9/9/8**	**2**/2	22/**18**	**9**/2	**3**	2.5/3.3	**3**	**10/16**
5	17	**CMT1a**	**21/27/17**	**23/NA/11**	NA	**34/22**	**15**	**3**	**3.5/4.2**	**4**	**12/12**
6	17	**CMT1b**	**32/15/20/17**	**27/12/13**	NA/3	30/**22**	**22**/3	3	**3.8/5.2**	**4**	**12/16**
7	16	CMT2	7/10/5/8	5/5/4	1/1	NA	NA	NA	2.0/2.5	2	0/12
8	11	CMT2a	8/8/7/8	6/7/6	2/2	21/6	6/3	2	2.0/2.8	2	0/17
9	8	CMT2e	5/4/3/6	4/4/3	2/1	12/8	6/2	2	2.0/2.7	2	0/17
10	8	***CMT4c***	**10**/8/4/7	5/5/**6**	2/2	23/3	**9**/2	**3**	**2.9/4.2**	2	**6/17**
11	11	***CMT4c***	NA	NA	NA	15/**12**	NA	1	**NA**	NA	**1/3**
12	10	***CMT4d***	**15/**8/7/6	**14/**10/7	2/2	17/**12**	7	2	**3.0/4.6**	2	**5/17**
13	13	***GAN***	6/**10**/**15**/**13**	7/**9**/**12**	1/1	**33/17**	4/3	2	2.0/3.4	2	**7/17**
		Acquired neuropathies									
14	15	***GBS***	11/10/7/9	**11**/7/**9**	1/1	25/**16**	7/3	3	2.1/3.7	**4**	**4/17**
15	10	***GBS***	7/9/7/**11**	5/5/5	1/1	19/**12**	7/2	2	2.3/**3.8**	**8**	**4/17**
16	17	***GBS***	7/6/7	5/NA/7		25/7	7	2	**2.9/4.2**	3	**2/12**
17	18^*^	***CIDP***	**16/15/**9	7/9/7	NA/**4**	**55/19**	**12/4**	**5**	**3.0/6.2**	**6**	**11/15**
18	13	***CIDP***	**22**/9/**9**/9	8/4/**12**	2/2	28	**8**/3	3	2.3/3.4	**3**	**5/16**
		Phakomatosis									
19	15	NF1	11/8/7/10	11/7/5	2/2	20/10	6/2	2	3.3/2.6	3	0/17
20	17	**NF1**	**69/78/23/17**	**52/19/37**	**NA**	**77/87**	**44/67**	**13**	**6.0/6.1**	**14**	**15/15**
		Storage diseases									
21	12	**MLD**	**13/10/11/13**	7/4/**8**	2/2	19/**16**	**10**/3	**7**	**3.3**/3.1	**3**	**10/17**
22	13	**Krabbe**	**25/16/11/15**	**10/14/**7	NA/**4**	31/**14**	**12**/7	2	2.7/**5.6**	3	**11/16**
23	14	***Maltase***	**20/17/**6**/12**	10/**10/9**	5/4	23/**13**	**10**/3	2	NA	2	**7/15**
		Other diagnoses									
24	11	None^*^	9/7/8/9	7/6/6	2/2	19/9	7/2	1	3.5/2.0	2	0/17
25	17	None^*^	12/10/5/5	9/4/4	1/1	22/9	6/2	2	3.5/2.5	2	0/17

Three patients have just reached their 18th since few weeks (<10 weeks).

* *Initially suggested neuropathy without pathology in clinical examination, neurophysiology and lab testing (pain syndrome and mild cognitive deficits of unknown reason). Nerve enlargements are in bold print, those patients with multifocal nerve enlargement (< half of the measurements enlarged) are additionally in italic, whereas those without nerve enlargement are unmarked. CMT, Charcot-Marie-Tooth hereditary neuropathy type 1, 2 or 4; CIDP, chronic inflammatory demyelinating neuropathy, GAN, giant axon neuropathy; GBS, Guillain-Barré syndrome; MLD, metachromatic leukodystrophy; NF, neurofibromatosis type1*.

### Discussion

Reference data are the basis for correct nerve ultrasound performance (1–8). With this data collection, we could describe normal values of teenagers and could show that nerves grow gradually during childhood (Figure 1). From approximately the age of 15 to 17, nerves will have reached their maximum size. The influence of BMI and gender on nerve size seems to be limited, according to our multivariate analyses. Side-to-side differences reached 1 to 5% percent in mean but could even reach up to 50% in some individuals. The intranerve CSA variability—an intraneural nerve ratio—was similar to those published for adults (7) and children <8 years (10) in the median, ulnar, and tibial nerve. By defining normal and upper limits (90. percentile), pathological generalized or multifocal nerve enlargement was found in children/adolscents with hereditary and acquired—mostly demyelinating—neuropathies.

**Figure 3:**
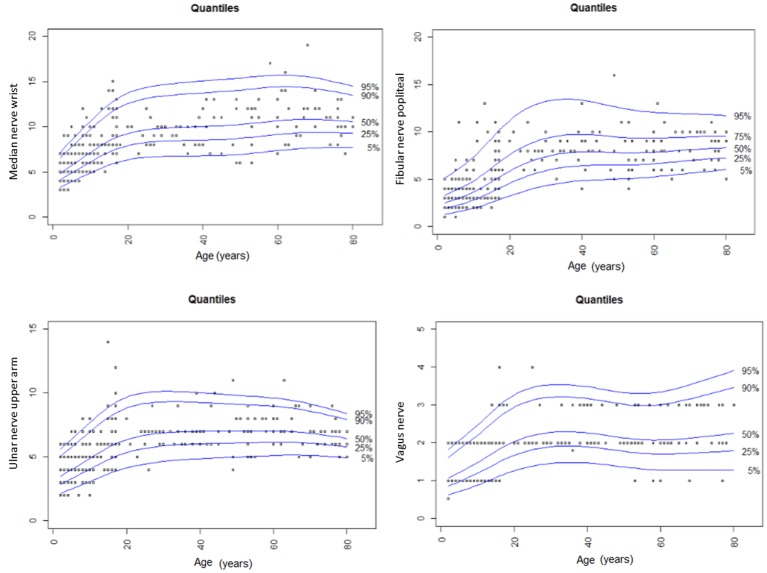
Summary of CSA findings of the median, the fibular, the ulnar and the vagus nerve in our study compared to values in children <8 years and adults of the same study group (9, 10). The nerve growth correlates with age until late adolescents and then reaches its maximum size in the young individual.

With regard to reference values of children <8 years (10), nerve growth correlates with age during the whole life span of the young; however, it seems to gain momentum in adolescents, according to values found in our own lab (Figures 1–3) and (10).

Thus, according to the obvious age dependency in adolescent nerve growth, normal values for each age were defined (Table 3) in contrast to infants and young children <8 years, where grouped values seemed to be sufficient (10). As both studies have been performed by the same author group using the same protocol and ultrasound device, a comparison of these data seems to be appropriate.

With regard to several published adult data, nerves in school children and adolescents are smaller until the age of 15 to 17 (4–9). At this age, then, the mean and the upper normal values are comparable to those in adults [Table 3, according to 9]. In adults, nerve growth seems not to correlate with age anymore except for some locations, i.e., the tibial nerve or the median nerve at the wrist (9).

It remains uncertain why nerve growth reaches a peak in young people and then even decreases again in seniority; however, one explanation might be reduced fluidity, reduced elasticity, and increased stiffness in the nerves of older people (Figure 3). Interestingly, the wrist CSA in the median nerve and other entrapment sites increases, and this is probably due to the higher rate of entrapment syndromes in old people. Even the vagus nerve seems to be enlarged with age, which might be a consequence of increasing autonomic dysfunction in the older humans. Similar findings have already been discussed before (17); however, longitudinal data are necessary for nerve size development during the life span. In our population, the influences of gender differences and BMI were negligible; data in literature are also inconsistent. Most authors thus decided to publish gender-, height-, and weight-independent reference values (4–10).

In a second step of this study, we compared our data to age-matched patients with a proven diagnosis of neuropathy as proof concept. As in adults (2, 4), children and adolescents with hereditary demyelinating neuropathy, e.g., CMT1 revealed generalized nerve enlargement according to our reference values. Similar data have already been published for younger children (18). Of note was the fact that many nerve segments would have been rated as normal if we had used adult normal values (9). In contrast, axonal CMT2 patients revealed no CSA enlargement. This finding is also in line with our knowledge from adults (2, 4, 19). For autosomal-recessive CMT4 types, literature is sparse, and the finding of partially enlarged nerves, fitting to the mostly demyelinating pathology, is therefore interesting and must be further validated. Children/adolescents with inflammatory neuropathies seem also to reveal proximal predominant nerve enlargement in chronic radiculoneuritis and root/vagus predominant nerve enlargement in acute inflammatory radiculoneuritis. This pattern is even quite similar to that described in adults and is in line with our limited knowledge of nerve imaging in children and adolescents (12–14).

Finally, some patients with very rare neuropathy types, e.g., lysosomal storage diseases [MLD, M. Krabbe, sulfatase deficiency] haven been examined. They even exhibited nerve enlargement, which might be a sign of intraneural accumulation of metabolites (20, 21). This finding is very promising, as it opens new doors to non-invasive diagnosis of rarer neuropathies in children/adolescents; so far, not much is known yet about lysosomal storage disease-associated neuropathies except for related disorders, e.g., adrenoleukodystrophy (14) or one case report of MLD (22), and further studies will thus be promising for the future. Children/adolescents without neuropathy had, overall, no CSA enlargement, which emphasizes the power of ultrasound to exclude neuropathies.

Moreover, in two patients with neurofibromatosis (NF) type 1, we could differentiate between one child with high peripheral tumor load and another without any peripheral tumor; this might underline the importance of ultrasound screening in the surveillance of patients with peripheral nerve sheath tumors, as also for adults the finding of all (nerves)-or-nothing has been described for NF1 (23).

The interpretation of our data must be done with care: (1) our data have been collected within Caucasian population and thus might be different in countries with predominantly other ethnicities; further reference studies are therefore needed. (2) Ultrasound devices might show differences concerning normal values. Each lab might therefore create their own normal data values, although we suggest the device-independent stability of CSA measurements to be high. (3) Our comparison between children <8 and >8, adolescents and adults has been carried out based on distinct study group data; nevertheless, the protocol and the device were the same as the standardized one, and examiners were blinded to each other. The interrater and the intrarater ICCs were excellent in all studies. (4) Our upper limits have been calculated by using the 90^th^ Percentile, which is a compromise solution between sensitivity and specificity. (5) Reference data for C5 and C6 in young children have to be interpreted with caution, as only a part of the participants was examined sufficiently at this part of the neck. We suggest that plexus parts, i.e., the trunks, might be easier to visualize, and, thus, further studies would be needed concerning the plexus in children. (6) We must confess that the number of lefthanded individuals was somewhat higher in the older group than in the younger; however, the overall influence of the handedness was not statistically significant in our study. (7) Overall conclusion concerning the ultrasound pattern in distinct neuropathies in children/adolescents is not possible out of these data due to the small sample sizes of the patient group. For the same reason, statistical analyses have not been carried out. Further studies concerning those entities are needed. (8) Echointensity and fascicle counting are of further interest in study ultrasounds; however, our device was not suitable enough to visualize those data in children and adolescents. Herein, ultrahigh-resolution probes might be helpful (24).

Alltogether, these data are the first normal data for a large cohort of children >7 years and adolescents. For each age, normal values seemed to be necessary as growing correlates significantly with aging. The influence of gender and BMI in our cohort was negligible. Side-to-side differences at some nerve landmark (1 to 5% in mean) must be kept in mind before defining pathology. These reference values simplify the analysis of children and adolescents with neuropathic disorders and clarify underlying diagnoses in many cases. These data enable initiations of nerve imaging studies in distinct pediatric neuromuscular disorders.

## Data Availability Statement

All datasets generated for this study are included in the article/supplementary material.

## Ethics Statement

The studies involving human participants were reviewed and approved by Tuebingen University Ethic committee. Written informed consent to participate in this study was provided by the participants' legal guardian/next of kin.

## Author Contributions

A-SG, CS, AG, NW, and SG were involved in the project development, the data acquisition, the data analysis and the drafting of the manuscript. J-HS, HK, VH, JK, and LS-H were involved in data acquisition and analysis. All authors carefully read the manuscript.

## Conflict of Interest

The authors declare that the research was conducted in the absence of any commercial or financial relationships that could be construed as a potential conflict of interest.

## Abbreviations

MN: median nerve
UN: ulnar nerve
RN: radial nerve
TN: tibial nerve
FN: fibular nerve
C5/6: cervical spinal nerve 5 and 6
VN: vagus nerve
SN: sural nerve
s: superficial
PIN: posterior interosseous nerve
PF: popliteal fossa
CSA: cross-sectional area
SD: standard deviation
CMT: Charcot-Marie-Tooth hereditary neuropathy type 1, 2, or 4, CIDP, chronic inflammatory demyelinating neuropathy
GAN: giant axon neuropathy
GBS: Guillain-Barré syndrome
MLD: metachromatic leukodystrophy
MHz: Megahertz
mm^2^: square millimeter
NF: neurofibromatosis
kg: kilogram
BMI: Body mass index.
